# Curcumin Enhances the Abscopal Effect in Mice with Colorectal Cancer by Acting as an Immunomodulator

**DOI:** 10.3390/pharmaceutics15051519

**Published:** 2023-05-17

**Authors:** Kuang-Chung Shih, Hui-Wen Chan, Chun-Yi Wu, Hui-Yen Chuang

**Affiliations:** 1 Division of Endocrinology and Metabolism, Department of Medicine, Cheng-Hsin General Hospital, Taipei 11220, Taiwan; shihkc19610909@gmail.com; 2 Division of Endocrinology & Metabolism, Tri-Service General Hospital, National Defense Medical Center, Taipei 11490, Taiwan; 3 School of Medicine, National Defense Medical Center, Taipei 11490, Taiwan; 4 Department of Biomedical Imaging and Radiological Sciences, National Yang Ming Chiao Tung University, Taipei 11221, Taiwan; viator70757@gmail.com (H.-W.C.); chunyiwu@nycu.edu.tw (C.-Y.W.)

**Keywords:** curcumin, abscopal effect, colorectal cancer, radiolabeling, OX40, immune, apoptosis

## Abstract

Radiotherapy (RT) is an effective cancer treatment. The abscopal effect, referring to the unexpected shrinkage observed in non-irradiated tumors after radiation therapy, is thought to be mediated by systemic immune activation. However, it has low incidence and is unpredictable. Here, RT was combined with curcumin to investigate how curcumin affects RT-induced abscopal effects in mice with bilateral CT26 colorectal tumors. Indium 111-labeled DOTA-anti-OX40 mAb was synthesized to detect the activated T cell accumulations in primary and secondary tumors correlating with the changes in protein expressions and tumor growth to understand the overall effects of the combination of RT and curcumin. The combination treatment caused the most significant tumor suppression in both primary and secondary tumors, accompanied by the highest ^111^In-DOTA-OX40 mAb tumor accumulations. The combination treatment elevated expressions of proapoptotic proteins (Bax and cleaved caspase-3) and proinflammatory proteins (granzyme B, IL-6, and IL-1β) in both primary and secondary tumors. Based on the biodistribution of ^111^In-DOTA-OX40 mAb, tumor growth inhibition, and anti-tumor protein expression, our findings suggest that curcumin could act as an immune booster to augment RT-induced anti-tumor and abscopal effects effectively.

## 1. Introduction

Approximately half of cancer patients receive radiotherapy (RT) in their treatment course, and RT induces cell death by producing DNA breaks. RT-mediated cell death has been shown to enhance anti-tumor immunity through increasing the levels of proinflammatory cytokines, tumor-associated antigens (TAAs), and damage-associated molecular patterns (DAMPs), which can trigger dendritic cell (DC) maturation and activation of antigen-specific CD8^+^ T cells [1]. RT-enhanced immune response named the “abscopal effect” has attracted much attention recently, although it is clinically rare [2]. The abscopal effect has effectively inhibited unirradiated tumors and metastatic lesions, and the treatment efficacy seems dose-dependent [3,4]. Azad et al. showed that RT turns nonimmunogenic pancreatic cancer into immunogenic by enhancing PD-L1 expression and T-cell infiltration in mice [5]. Besides, Carbon ion therapy has been proven to induce abscopal effect more efficiently and profoundly than photon therapy, resulting in more substantial cell damage, especially when combined with immune checkpoint inhibitors (ICIs) [6]. In contrast, RT can also make the tumor microenvironment (TME) more immunosuppressive by enhancing angiogenesis [7], triggering M2 macrophage polarization [8], and resulting in tissue fibrosis by activating the TGF-β and WNT/catenin pathways [9,10,11]. Increased TGF-β levels and tumor-associated macrophages in the TME are correlated with higher recurrence and metastatic rates after RT. The correlations between the given RT doses and TME reconstruction remain conflicting [12,13]. Nevertheless, treating patients with an “optimal” RT dose is clinically irrelevant in generating a less immunosuppressive TME and costs overall tumor control.

Rather than adjusting the radiation dose, combining immunomodulators with RT might be a superior alternative to improve the current situation. Curcumin has been shown to exert anti-tumor and immunomodulatory properties in several studies. Curcumin suppresses tumor growth by generating reactive oxygen species (ROS) and inducing apoptosis. Curcumin triggers apoptosis in HCT-116 cells by inhibiting NF-κB activation and downstream pathways [14]. Curcumin has been found to cause more DNA damage and cell deaths in Panc-1 and MiaPaCa-2 cells when combined with RT [15]. Additionally, curcumin exerts immunomodulation potential and slows tumor progression [16,17]. For instance, curcumin augments tumor inhibition by enhancing the proliferation and activation of T cells [18]. Curcumin can also suppress tumor growth by repressing the generation and activity of TGF-β-induced regulatory T cells (Tregs) [19]. Thus, RT outcomes may be augmented when combined with curcumin by modulating the immune TME. Therefore, we used curcumin as an immunomodulator to enhance RT outcomes in a bilateral tumor-bearing mouse model in the current study. Immunology-related studies commonly utilize many living subjects to observe the changes in vivo instead of studying on the same cohort longitudinally, due to traditional techniques often requiring euthanizing animals to analyze the immune populations in the specific tissues and organs. Molecular imaging (MI) has been developed for decades and helps visualize various biological processes from the molecular scale to the whole animal scale. Hence, MI has been widely applied in cancer research and immunology studies because studies can be conducted using the same animal cohorts to examine the changes resulting from different treatments longitudinally [20]. Like CD25, OX40 is a T-cell activation marker, implying better treatment outcomes [21]. Here, we radiolabeled anti-OX40 monoclonal antibody (mAb) with ^111^In to observe the accumulation of activated T cells in tumor-bearing mice receiving different treatments by conducting biodistribution assays. The results were further correlated with the tumor growth, CD4^+^ and CD8^+^ T cell frequencies in primary tumor-drainage lymph node (TDLN) and spleen, and the changes in apoptosis- and immune-related proteins.

Currently, limited studies have investigated how curcumin influences the RT-induced abscopal effect and related immunomodulation. In the present study, we validated that curcumin can function as an immunomodulator and boost the RT-induced abscopal effect by increasing apoptosis and reverting the immunosuppressive TME in a bilateral CT26 tumor-bearing mouse model.

## 2. Materials and Methods

### 2.1. Cell Line, Drug Preparation, and Cytotoxicity Assay

The murine colorectal cancer cell line CT26 was maintained in the RPMI 1640 medium containing 10% fetal bovine serum and 1% P/S and kept in a 37 °C humidified incubator with 5% CO_2_. Curcumin purchased from Sigma-Aldrich (C1386, St. Louis, MO, USA) was dissolved in DMSO to make a stock solution with a concentration of 40 mM. The stock solution was kept at −20 °C for further use.

CT26 cells were seeded into a 96-well plate at a density of 2 × 10^4^ cells/well a day before being treated with different concentrations (0, 2.5, 5, 25, 37.5, 50, 75 µM) of curcumin for 24 h. The concentration of DMSO for all the treated groups, including the vehicle control group, was 0.1%. Cell viability was determined by conducting 3-(4,5-dimethylthiazol-2-yl)-2,5-diphenyltetrazolium bromide (MTT) assays. Briefly, the cells were cultured in 0.5 mg/mL MTT-containing medium for 3–4 h after drug removal. Purple formazan was dissolved with DMSO, then the plate was read at 570 nm. Cell viability was calculated to obtain the IC_50_ value using the following formula:

$$cell\;viability\left(\%\right)=\frac{{OD}_{570}\;of\;the\;treated\;group}{{OD}_{570}\;of\;the\;vehicle\;control\;group}\times 100\%$$

### 2.2. Colony Formation Assay

Various numbers of CT26 cells were seeded into 6 cm dishes after being irradiated with 0–8 Gy X-ray using a cabinet irradiator (RS2000, Rad Source, Buford, GA, USA). Cells were stained with crystal violet and observed using a dissecting microscope on day 10 after incubation. Only the colonies with >50 cells were counted and used for calculating the surviving fraction. The radiation survival curve was generated by plotting the X-ray doses against the corresponding surviving fraction. The surviving fraction was calculated using the following formula:

(1) $$surviving\;fraction\;at\;a\;given\;dose=\frac{number\;of\;colonies\;counted/numbers\;of\;cells\;seeded}{plating\;efficiency\;of\;the\;non-irradiated\;cells}.$$

### 2.3. Therapeutic Evaluation of the Combinational Treatment of Curcumin and Radiotherapy in a Bilateral Tumor-Bearing Mouse Model

**Figure 1:**
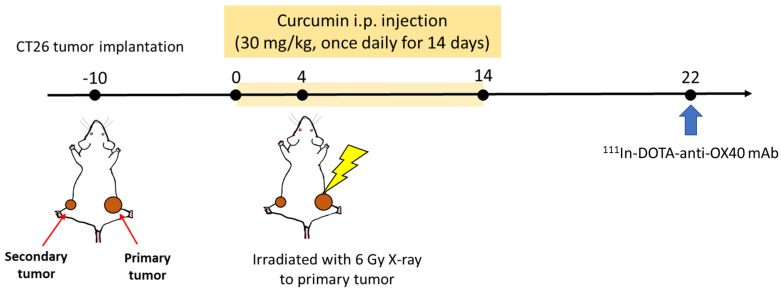
In vivo experimental scheme for evaluating treatment efficacy and abscopal effect on a bilateral tumor-bearing model.

All the animal experiments and procedures were approved by the Institutional Animal Care and Use Committee of National Yang Ming Chiao Tung University (approval 1091106n). A bilateral CT26-bearing mouse model was established to study the abscopal effect after combining radiotherapy with curcumin treatment. As shown in Figure 1, 5 × 10^5^ and 3 × 10^5^ cells/100 µL PBS were implanted on the right and left flanks of 6-week-old female BALB/c mice (National Laboratory Animal Center, Taipei City, Taiwan) to create the primary and the secondary tumors, respectively. Tumor growths were monitored by caliper measurement to calculate the tumor volume twice a week by the equation:

$$Tumor\;volume\left({mm}^{3}\right)=0.5\times length\times {width}^{2}$$

Tumor-bearing mice were grouped as follows when the average volume of primary tumors reached 100 mm^3^, including control (CTRL), curcumin (CUR), radiotherapy (RT), and combination (COMB), to perform the subsequent therapeutic studies, with N = 4 per group and all animal experiments repeated twice. Mice in the curcumin and combination groups received curcumin (30 mg/kg) by intraperitoneal (IP) injection once per day for 14 consecutive days. Mice in the control and RT groups also received an equal volume of PBS through IP injection for 14 days. Mice in the RT and combination groups were irradiated with 6 Gy X-ray on day 4 after the first curcumin treatment. Only the primary tumors received irradiation, as the other parts of the body were properly shielded. Tumor growths were observed for 21 days. At the end of the study, lymph nodes, spleens, and tumor tissues were harvested and subjected to flow cytometry, Western blot, and ELISA to investigate the potential immunomodulation effects.

### 2.4. Flow Cytometry—Frequencies of CD4^+^ and CD8^+^ T Cells

Spleen and tumor-draining lymph nodes (of the primary tumor) were harvested from the tumor-bearing mice on day 21 and pressed through 70 µm cell strainers using the plungers of 3 mL syringes. Cell suspensions were treated with red blood cell (RBC) lysis buffer to remove red blood cells before being stained with the antibodies listed below. After washing, the frequencies of CD4^+^ and CD8^+^ T cells in cell suspensions were determined by flow cytometry (Beckman, CytoFLEX, Coulter, CA, USA).

### 2.5. Radiolabeling of Indium-111 Anti-OX40 mAb

Prior to the indium-111 radiolabeling, anti-OX40 mAb (#BE0031, clone OX86, BioXCell, Lebanon, NH, USA) was resuspended in PBS at 0.5 mg/mL and then incubated with fivefold excess DOTA-NHS ester (#HY-128890, MedChemExpress, Monmouth Junction, NJ, USA) at 4 °C overnight. Excess DOTA-NHS ester was removed using 3 kDa desalting columns (#28-9322-18, GE Healthcare, Chicago, IL, USA), and the remaining product was suspended in PBS and stored at 4 °C.

^111^InCl_3_ (1 mCi) was reacted with 100 µg DOTA-anti-OX40 mAb in HEPES buffer (pH 4.5, 0.1 M) at 37 °C for an hour with gentle shaking, and 0.1 M DTPA was added to a final concentration of 0.01 M and incubated at room temperature for 30 min to scavenge unchelated ^111^In ions. The reactants were purified using desalting columns (3 kDa) and washed with normal saline thrice. The resulting^111^In-DOTA-anti-OX40 mAb was suspended in PBS and stored at 4 °C. The radiolabeling efficiency and radiochemical purity of ^111^In-DOTA-OX40 mAb were determined by radio-TLC using 0.5 M sodium citrate (pH 4.5) as mobile phase. The ^111^In-DOTA-OX40 mAb remained at the origin (Rf 0–0.2), while the unbound ^111^In and ^111^In-citrate migrated to the solvent front (Rf 0.85–1.05).

### 2.6. Biodistribution of ^111^In-DOTA-Anti-OX40 mAb

Mice were euthanized 48 h after being intravenously injected with 50 µCi/10 µg of ^111^In-DOTA-anti-OX40 mAb on day 21. Designated organs or tissues were collected, and the radioactivity in each sample was measured by a gamma counter. The corrected reading was further divided by the weight of each sample, and the results are shown as percentage injected dose (%ID)/g to realize the biodistribution of ^111^In-DOTA-anti-OX40 mAb in bilateral tumor-bearing mice receiving various treatments.

### 2.7. Western Blot

For in vitro Western blot, CT26 cells were treated with different doses of curcumin for 24 h, then collected and lysed by RIPA buffer on ice. For in vivo Western blot, harvested tumors were incubated with RIPA buffer and sonicated for 30 sec on ice, then 30 µg of total protein was loaded in each well and separated by 10% or 12% SDS-PAGE. Proteins were then transferred to polyvinylidene difluoride (PVDF) membranes and blocked by 5% non-fat dry milk or 5% BSA to reduce non-specific binding. Next, the membranes were incubated with primary antibodies against the proteins of interest listed below overnight at 4 °C. Membranes were washed and incubated with secondary antibodies before signal acquisition. Finally, the signals were detected by an enhanced chemiluminescence reagent (#LF08-500, Visual Protein, Taipei, Taiwan) using the ImageQuant™ LAS 4000 system. The band intensity was quantified through ImageJ (version 1.53, NIH, Bethesda, MD, USA), and β-actin was utilized as the loading control. Proteins of interest were NF-κB, TNF-α, COX-2, PD-L1, TGF-β, granzyme B, IL-1β, IL-6, Bcl-2, BAX, and cleaved caspase-3. All the antibodies were purchased from Genetex (Hsinchu City, Taiwan) or Cell Signaling Technology (Danvers, MA, USA).

### 2.8. ELISA

Tumor lysates from primary and secondary tumors of each group were subjected to ELISA to evaluate the changes in expressions of cytokines IL-1β (#432604), IL-6 (#431304), and IFN-γ (#430804). All the ELISA kits were purchased from Biolegend (San Diego, CA, USA). The manufacturer’s protocols were followed to perform the ELISA. The plates were read at 450 nm using a spectrometer (Infinite^®^200 PRO, TECAN, Männedorf, Switzerland). The concentration of each sample was determined by interpolating the standard curve.

### 2.9. Statistics

All the results represent mean ± standard error of the mean (SEM). Statistical analysis was performed using GraphPad Prism 9 software (GraphPad Software Inc.; San Diego, CA, USA). One-way ANOVA was used for multiple comparisons in biodistribution, flow cytometry, Western blot, and ELISA. Two-way ANOVA was conducted to compare the differences in tumor growth among all groups over time. Both one-way and two-way ANOVA was followed by the Tukey test, with *p* < 0.05 considered statistically significant. All the in vitro experiments were performed at least three times, and the in vivo experiments were performed twice.

## 3. Results

### 3.1. Curcumin Shows the Potential for Immunomodulation

**Figure 2:**
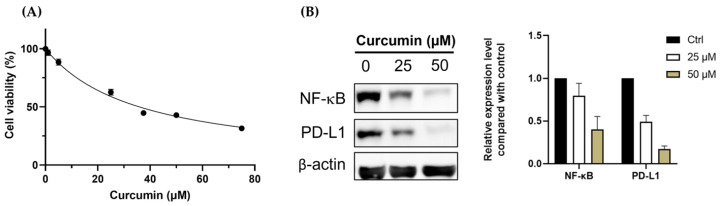
Curcumin suppresses the growth of CT26 murine colorectal cancer cells by inhibiting NF-κB and PD-L1 expressions. (**A**) The IC50 of curcumin in CT26 cells was determined by MTT assay, and was 50 µM. (**B**) Expression of NF-κB and PD-L1 were determined by Western blot after cells were treated with different doses of curcumin for 24 h. The quantified results were obtained by dividing the band intensity of the protein of interest (NF-κB and PD-L1) by the intensity of actin. All the values were normalized with the Ctrl (0 µM) group.

The cytotoxicity of curcumin in CT26 cells was determined by the MTT assay after 24 h incubation, and the IC50 of curcumin in CT26 cells was 50 µM (Figure 2A). NF-κB is one of the significant determinants of treatment resistance against radiotherapy and immune attack. Moreover, NF-κB has been demonstrated to regulate the PD-L1 expression directly. Therefore, we analyzed the expressions of both NF-κB and PD-L1 with Western blot. CT26 cells treated with 0.1% DMSO, ½ IC50, and IC50 of curcumin for 24 h were subjected to Western blot. Figure 2B shows that curcumin dose-dependently reduced the expressions of NF-κB and PD-L1, highlighting its potential as an immunomodulatory agent for cancer therapy.

### 3.2. Curcumin Enhances the Therapeutic Efficacy of Radiotherapy and Augments the Radiotherapy-Induced Abscopal Effect in Bilateral CT26 Tumor-Bearing Mice

**Figure 3:**
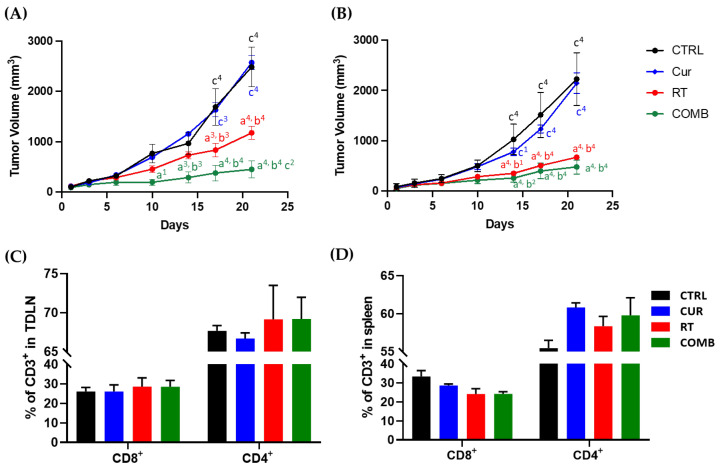
Tumor inhibition effects and the changes in the T cell frequencies detected in the tumor-draining lymph node (TDLN) and spleen caused by treatments. Growth curves of the (**A**) primary and (**B**) secondary tumors from day 1 to day 22 after treatment. At the end of the study, mice were euthanized for tissue harvesting, and the CD8 and CD4 T cell frequencies in the (**C**) TNLD and (**D**) spleen were determined using flow cytometry. a, compared with the CTRL group; b, compared with the Curcumin group; c, compared with the RT group; 1, *p* < 0.05; 2, *p* < 0.01; 3, *p* < 0.001; 4, *p* < 0.0001.

The primary tumor growth curves (Figure 3A) reveal no difference in tumor sizes detected between the CTRL and CUR groups at all times, indicating that 30 mg/kg curcumin could not suppress the CT26 tumor in vivo. However, the RT group showed moderate tumor inhibition, and the COMB group significantly suppressed tumor growth compared to the CTRL and CUR groups from day 10 after treatment. It is worth noting that a significant difference (*p* < 0.01) in tumor size was also detected between the RT and COMB groups on Day 21.

Interesting findings were observed in the secondary tumors, as shown in Figure 3B: 30 mg/kg slightly slowed the growth of the secondary tumors in mice; however, no significant difference was detected compared to the CTRL group. The radiotherapy-induced abscopal effect was observed in the RT group from Day 17, as the average size of secondary tumors was significantly smaller than the CTRL group (*p* < 0.001). The average secondary tumor size in the RT group was also considerably smaller than the CUR group at the last time point (*p* < 0.001). Most importantly, the combination of curcumin and RT also resulted in the most significant inhibition of the secondary tumors. Considerable significance was detected from day 14 and day 17 compared to the CTRL and CUR groups. Even though the COMB group seemed to have the most profound suppression of the secondary tumor among all the groups, no significant difference was detected compared to the RT group until the last time point. The in vivo therapeutic evaluation results indicate that 30 mg/kg curcumin was able to enhance the RT-mediated anti-tumor effects and boost the RT-induced abscopal effect and overall anti-tumor immune responses.

Tumor-drainage lymph nodes and spleens were harvested and analyzed by flow cytometry on day 21 to understand whether the differences in tumor inhibition were related to T cell-mediated immunity. As shown in Figure 3C, 6 Gy RT and combination treatment increased the frequencies of CD8^+^ and CD4^+^ T cells compared to the CTRL group without resulting in a significant difference. On the other hand, all treated groups showed a slight reduction and increment in splenic CD8^+^ and CD4^+^ T cells, respectively (Figure 3D).

### 3.3. Biodistribution of ^111^In-DOTA-Anti-OX40 mAb Reveals That the Combination Treatment Led to Higher Activated T Cell Accumulation in the Primary and Secondary Tumors

**Figure 4:**
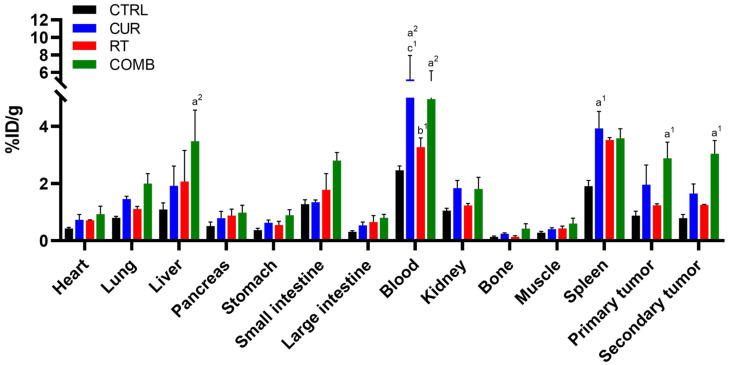
Biodistribution results of ^111^In-anti-OX40 mAb in mice receiving different treatments. Mice were euthanized 48 h after being injected with ^111^In-anti-OX40 mAb, designated organs/tissues were collected, and their radioactivity was counted using a gamma counter. Except for blood, the liver, spleen, and tumors showed higher relative radioactivity than other organs. The COMB group had the highest spleen and tumor uptakes among all the groups. a, compared with the CTRL group; b, compared with the Curcumin group; c, compared with the RT group; 1, *p* < 0.05; 2, *p* < 0.01.

The scheme of radiolabeling of ^111^In-DOTA-anti-OX40 mAb is shown in Supplementary Figure S1A, and the radiolabeling efficacy and purity are shown in Supplementary Figure S1B,C. The biodistribution results of ^111^In-DOTA-anti-OX40 mAb in bilateral CT26 tumor-bearing mice receiving various treatments are summarized in Supplementary Table S1. The quantitative results are shown in Figure 4. The blood radioactivity remained high because of the prolonged circulation time of whole antibodies, and minimal radioactivity was detected in bone and muscle for all groups. Expect for spleens and tumors, the accumulations of ^111^In-DOTA-anti-OX40 mAb in other organs and tissues were comparable with no significant difference among all groups. All treated groups had significantly higher accumulations of ^111^In-DOTA-anti-OX40 mAb in the spleens than the CTRL group. The ^111^In-DOTA-anti-OX40 mAb in primary and secondary tumors were also elevated in all treated groups compared to the CTRL group, indicating a potential immunity-boosting effect provoked by 30 mg/kg curcumin and 6 Gy RT treatments. The COMB group showed significant increases in the tumor accumulation of ^111^In-DOTA-anti-OX40 mAb compared to the CUR and RT groups, indicating that the combination treatment may elicit a more robust anti-tumor immune response. Interestingly, the secondary tumors in all groups exhibited comparable ^111^In-DOTA-anti-OX40 mAb accumulations to their corresponding primary tumors.

### 3.4. Combination Treatment Upregulates Proinflammatory, Proapoptosis-Associated Protein Levels and Elevates the Concentrations of Proinflammatory Cytokines in Tumors

**Figure 5:**
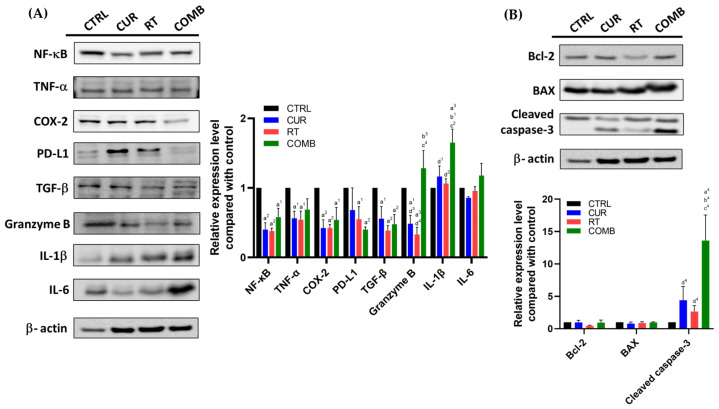
Protein changes in the primary tumors receiving different treatments. The tumors were collected, lysed, and subjected to Western blot to assess the changes in (**A**) NF-κB, its related proteins, and other immune-related proteins, including TGF-β, granzyme B, IL-1β, and IL-6, (**B**) Bcl-2, BAX, and cleaved caspase-3 expression. a, compared with the CTRL group; b, compared with the Curcumin group; c, compared with the RT group; d, compared with the COMB group; 1, *p* < 0.05; 2, *p* < 0.01; 3, *p* < 0.001; 4, *p* < 0.0001.

**Figure 6:**
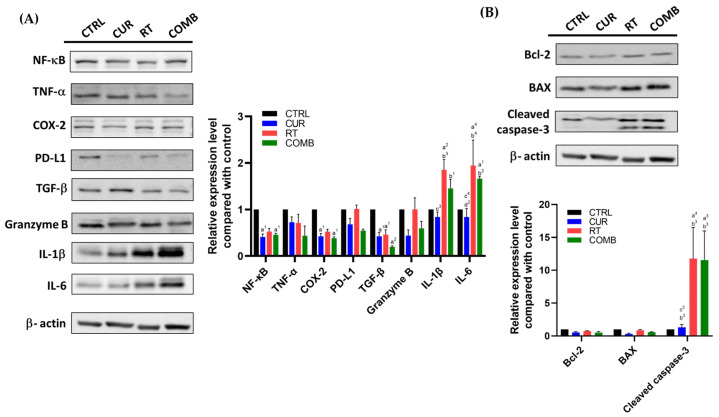
Protein changes in the secondary tumors from the mice receiving different treatments. The tumors were collected, lysed, and subjected to Western blot to assess the changes in (**A**) NF-κB, its related proteins, and other immune-related proteins, including TGF-β, granzyme B, IL-1β, and IL-6, (**B**) Bcl-2, BAX, and cleaved caspase-3 expression. Note: Secondary tumors did not receive RT and were used to investigate the potential abscopal effect. a, compared with the CTRL group; b, compared with the Curcumin group; c, compared with the RT group; d, compared with the COMB group; 1, *p* < 0.05; 2, *p* < 0.01; 3, *p* < 0.001; 4, *p* < 0.0001.

Tumor lysates were analyzed by Western blot and ELISA to uncover the underlying molecular mechanism leading to tumor inhibition. As mentioned, NF-κB is strongly related to the resistance developed against radiotherapy and immune attack. The NF-κB levels were found to decrease in all treated groups compared to the CTRL group. Its downstream proteins, such as TNF-α, COX-2, and PD-L1, were also reduced in all treated groups. On the other hand, proinflammatory cytokines IL-1β and IL-6 were increased in the primary tumors of the COMB group (Figure 5A). Figure 5A also indicates that the combination treatment elevated granzyme B expression in the primary tumors, which aligns with the significant increase in cleaved caspase-3 level shown in Figure 5B. Similar results were also observed in the secondary tumors (Figure 6A,B), except for the reduction in granzyme B detected in the COMB group. However, both IL-6 and IL-1β levels were still elevated in the RT and COMB groups, which might have resulted in the higher cleaved caspase-3 level and better tumor inhibition seen in these two groups.

**Figure 7:**
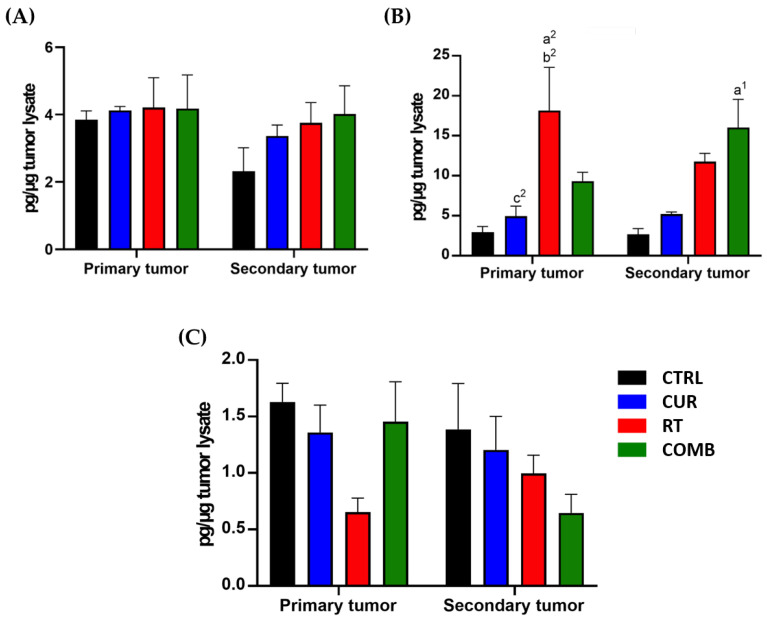
ELISA determines expressions of proinflammatory cytokines in both tumors. Levels of proinflammatory cytokines, including (**A**) IL-1β, (**B**) IL-6, and (**C**) IFN-γ, were measured using ELISA. Except for IFN-γ, all the treatments increased IL-1β and IL-6 expression to different degrees. a, compared with the CTRL group; b, compared with the Curcumin group; c, compared with the RT group; 1, *p* < 0.05; 2, *p* < 0.01.

The concentrations of IL-1β, IL-6, and IFN-γ in the primary and secondary tumors determined by ELISA are shown in Figure 6. IL-1β is known to enhance proinflammatory and anti-tumor effects and help the recruitment of immune cells, and IL-6 can be produced by various immune cells and shows both proinflammatory and anti-inflammatory roles according to the surrounding environment. Thus, evaluating the IL-6 change caused by different treatments and correlating them with the treatment responses is critical. The trends of IL-1β and IL-6 results (Figure 7A,B) were like those observed by Western blot. IFN-γ is a well-known anti-viral cytokine that can regulate both innate and adaptive immunity. Although many studies have indicated that IFN-γ can augment NK and T cell function and improve the anti-tumor responses, some recent studies also pointed out that IFN-γ may stimulate immunosuppression by empowering the synthesis of indoleamine-2,3-dioxygenase (IDO) and other immune checkpoint inhibitory molecules. Accordingly, we also measured the IFN-γ level and found it was suppressed in primary and secondary tumors after treatments (Figure 7C).

## 4. Discussion

Radiotherapy (RT) is a widely used cancer treatment that utilizes high-energy ionizing radiation to induce DNA damage, leading to cell death. The dying cells and cellular debris generated by RT can be taken up by surrounding antigen-presenting cells (APCs), including dendritic cells (DCs) and macrophages, then be present to T cells, initiating systemic immune responses. Activated immunity helps eradicate the remaining cancer cells expressing the same tumor antigens, resulting in the shrinkage of non-irradiated tumors, known as the “abscopal effect.” Although the abscopal effect is appealing, it is clinically rare and unpredictable. Strategies to enhance its incidence and magnitude have been heavily studied, but the success of such combination therapies is highly dependent on the immunogenicity of the tumor and the activation of the immune system. Therefore, identifying agents that can enhance the immune response is crucial for the success of combination therapy.

We chose curcumin for combination treatment due to its anti-tumor and immunomodulatory abilities. The IC50 of curcumin in CT26 murine colorectal cancer cells was 50 µM (Figure 2A). Treating the CT26 cells with 1/2 IC50 and IC50 significantly repressed the expressions of NF-kB and PD-L1, which are critical regulators of tumor progression and immune response (Figure 2B). Curcumin has been found to repress the expression of NF-κB and PD-L1. Liao et al. reported that curcumin suppresses PD-L1 levels and improves the tumor immune microenvironment in tongue squamous cell carcinoma [22]. To investigate the impact of curcumin on the RT-induced abscopal effect, a bilateral tumor-bearing model was established using BALB/c mice (Figure 1). This model is widely used to study the systemic anti-tumor effects of combination therapy [23]. The results showed that combining curcumin with RT resulted in the most significant tumor inhibition in primary and secondary tumors (Figure 3A,B). This finding suggests that curcumin has the potential to enhance the abscopal effect and improve treatment outcomes.

Curcumin has been found to improve the effectiveness of different treatments in various cancers. For example, Hussain et al. summarized that curcumin can augment the efficacy of cisplatin and reduce treatment resistance by increasing ROS generation and triggering apoptosis cascades in multiple cancer types [24]. A combination of curcumin and doxorubicin has also been shown to help tumor control by disturbing cell cycle progression and the balance between apoptosis and anti-apoptosis in head and neck cancer [25]. Curcumin has also been combined with FLLFOX chemotherapy for treating patients with metastatic colorectal cancer, resulting in significant improvement in overall and progression-free survival. However, further research is needed to fully elucidate the mechanisms underlying the beneficial effects of curcumin in cancer treatment [26]. Curcumin enhances radiosensitivity by regulating epigenetics, DNA damage repair, and apoptosis in various cancers, including nasopharyngeal cancer [27], bladder cancer [28], and glioblastoma [29]. Curcumin can also enhance radiotherapy through anti-inflammatory mechanisms [30]. Additionally, curcumin may enhance radiotherapy through anti-inflammatory mechanisms.

As curcumin possesses immunomodulatory properties and affects the NF-κB [31,32] and PD-1/PD-L1 [33] pathways, which are critical for T cell-mediated cell killing, we investigated the impacts caused by treatments on T-cell frequencies in the primary tumor draining lymph nodes (TDLN) and spleens in the tumor-bearing mice. While treatments did not increase CD8^+^ T cell frequency, they slightly increased CD4^+^ T cells in TDLN and significantly increased CD4^+^ T cells in spleens, as demonstrated by Figure 3C,D. OX40 is one of T cell activation markers, and its costimulatory role has been identified [34]. Alam et al. demonstrated that OX40 expression strongly correlates with positive tumor inhibition [21]. Therefore, we radiolabeled anti-OX40 mAb with indium-111 and used it to evaluate the status of T cell activation in vivo. Figure 4 indicates high accumulations of ^111^In-anti-OX40 mAb in the spleens, primary and secondary tumors, liver, and blood. The higher splenic signals detected in the treatment groups echoed the flow cytometry results, meaning that the treatments enhanced both the frequencies and functions of T cells in spleens. These results imply that these treatments may augment long-term anti-tumor immunity, as the OX40 level is tightly related to the survival of memory CD4^+^ and CD8^+^ T cells [34]. These results are consistent with changes in TDLN and splenic T cell frequencies reported in the tumor-bearing mice receiving other types of therapy, such as cryo-thermal therapy [35] and immunostimulatory therapy [36]. Moreover, the increased ^111^In-anti-OX40 mAb signals in blood suggest that T cells may be trafficking from the spleen to remaining tumor sites via the bloodstream, as CD8^+^ T cell frequency was found to be slightly decreased in the spleens but increased in the TDLNs.

Curcumin has been shown to enhance treatment outcomes by suppressing NF-κB as discussed; therefore, we analyzed the protein changes in the primary and secondary tumors using Western blotting and ELISA at the end of experiment. Figure 5 and Figure 6 show that NF-κB and its downstream proteins were suppressed by curcumin, RT, and combination treatment in both tumors. These results further confirmed the in vitro findings detected after curcumin treatment. Most importantly, the COMB group showed the highest expression of granzyme B, IL-1β, IL-6, and cleaved caspase-3 in the primary tumor, matching the tumor growth curve result. In the secondary tumors, RT and combination treatment led to upregulation of these four proteins, replicating the corresponding tumor growth. Reversing the immunosuppressive tumor microenvironment and augmenting T cell function by curcumin has been reported in multiple cancers, including tongue squamous cell carcinoma [22], head and neck cancer [37], and lymphoma [38]. TGF-β was also repressed by treatments compared to the CTRL group, potentially explaining the higher ^111^In-anti-OX40 mAb tumor accumulations in treated groups. Upregulated TGF-β can lead to an immunosuppressive tumor microenvironment [39], prompt metastasis, and cancer stemness [40]. Horn et al. also reported that blockage of PD-L1 and TGF-β simultaneously can improve anti-tumor immunity by increasing T cell infiltration and M1 tumor-associated macrophage (TAM) differentiation [41].

Elevated IL-1β and IL-6 expression in both tumor types (Figure 7A,B) indicated improved anti-tumor immunity with RT and combination treatment. However, unexpectedly, IFN-γ expression decreased in both tumors of all treated groups, especially in the secondary tumors (Figure 7C). IFN-γ is typically considered an anti-tumor cytokine and was expected to increase in tumors after treatment. The dark side of IFN-γ has been proposed and reviewed [42,43]. Its interaction with immunosuppressive cytokines such as TGF-β can lower effector T cell functions through upregulating the PD-1/PD-L1 pathway, enhancing Treg development and inhibiting cancer apoptosis. These may help explain the decreased IFN-γ expression along with the better tumor inhibition and tumor accumulation of activated T cells observed in this study.

Our findings demonstrate that curcumin enhances the abscopal effect in a bilateral CT26 tumor-bearing mouse model by suppressing NF-κB and downstream proteins, elevating IL-1β and IL-6, and modulating the overall tumor immune microenvironment, thereby enhancing tumor suppression. We also validated that curcumin enhances RT outcomes and boosts the anti-tumoral immune responses in bilateral CT26-luc tumor-bearing mice by performing a biodistribution study of ^111^In-DOTA-anti-OX40 mAb, ELISA assay, and Western blot. These results suggest that curcumin acts as an immunomodulator to improve RT outcomes. In future studies, we plan to use clinically relevant animal models and molecular imaging techniques to further investigate the dynamic changes in activated T cell distribution and correlate the in vivo and ex vivo findings to fully understand the boosted abscopal effect caused by cotreatment with curcumin or other molecules.

## Data Availability

The data generated and/or analyzed during the current study are available from the corresponding author on reasonable request.

## Supplementary Materials

The following supporting information can be downloaded at https://www.mdpi.com/article/10.3390/pharmaceutics15051519/s1. Figure S1: Scheme of radiolabeling procedure and the characterization of 111In-DOTA-anti-OX40 mAb; Table S1: Biodistribution results of 111In-DOTA-anti-OX40 mAb obtained from mice with bilateral CT26 tumors.
