# Supplementary material for: Curcumin Enhances the Abscopal Effect in Mice with Colorectal Cancer by Acting as an Immunomodulator
Source: Pharmaceutics. 2023 May 17;15(5):1519. doi: 10.3390/pharmaceutics15051519 (PMC10220687; doi:10.3390/pharmaceutics15051519)
Supplement: Supplementary file 1 [file pharmaceutics-15-01519-s001.zip › pharmaceutics-2383657-supplementary.pdf]

## Supplementary Materials

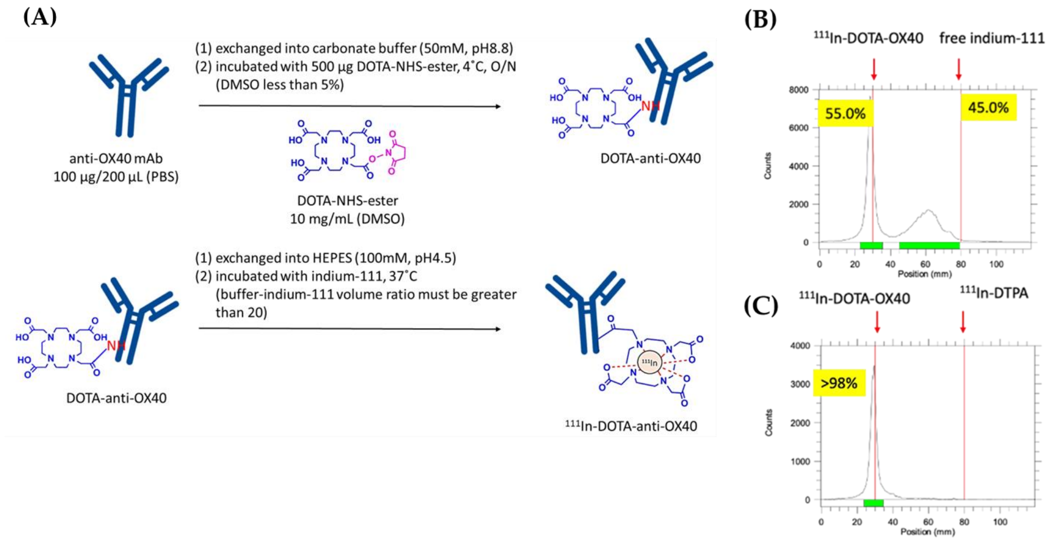


**Figure S1.** Scheme of radiolabeling procedure and the characterization of $^{111}$In-DOTA-anti-OX40 mAb.

**Table S1.** Biodistribution results of $^{111}$In-DOTA-anti-OX40 mAb obtained from mice with bilateral CT26 tumors.

| | Control | Curcumin | RT | COMB |
|---|---|---|---|---|
| Heart | 0.43 ± 0.06 | 0.73 ± 0.32 | 0.71 ± 0.04 | 0.93 ± 0.48 |
| Lung | 0.80 ± 0.09 | 1.46 ± 0.16 | 1.11 ± 0.16 | 2.00 ± 0.60 |
| Liver | 1.09 ± 0.40 | 1.92 ± 1.19 | 2.07 ± 1.88 | 3.48 ± 1.88 |
| Spleen | 1.91 ± 0.34 | 3.93 ± 1.02 | 3.52 ± 0.15 | 3.58 ± 0.58 |
| Pancreas | 0.51 ± 0.25 | 0.79 ± 0.41 | 0.88 ± 0.39 | 0.98 ± 0.45 |
| Kidney | 1.05 ± 0.15 | 1.84 ± 0.46 | 1.23 ± 0.13 | 1.81 ± 0.71 |
| Stomach | 0.37 ± 0.11 | 0.63 ± 0.16 | 0.55 ± 0.22 | 0.89 ± 0.34 |
| Small Intestine | 1.28 ± 0.26 | 1.35 ± 0.13 | 1.78 ± 0.98 | 2.80 ± 0.49 |
| Large Intestine | 0.31 ± 0.07 | 0.54 ± 0.20 | 0.65 ± 0.39 | 0.80 ± 0.21 |
| Blood | 2.46 ± 0.27 | 5.21 ± 4.73 | 3.27 ± 0.56 | 4.95 ± 2.16 |
| Bone | 0.13 ± 0.06 | 0.24 ± 0.06 | 0.14 ± 0.06 | 0.42 ± 0.30 |
| Muscle | 0.28 ± 0.08 | 0.40 ± 0.09 | 0.43 ± 0.14 | 0.60 ± 0.33 |
| Primary tumor | 0.88 ± 0.26 | 1.96 ± 1.19 | 1.24 ± 0.09 | 2.88 ± 0.98 |
| Secondary tumor | 0.79 ± 0.22 | 1.65 ± 0.58 | 1.25 ± 0.04 | 3.04 ± 0.80 |

Numbers represent mean values in %ID/g from three mice in each group.
